# Feasibility, Adoption, and Utility of Augmented Reality in Lumbar Puncture Simulation by Medical Students: A Pilot Study

**DOI:** 10.1089/jmxr.2024.0001

**Published:** 2024-07-30

**Authors:** Korak Sarkar, Jeffrey Coote, Rahim Abdul, Alec Slayden

**Affiliations:** ^1^ Ochsner Neurosciences Institute, Ochsner Health, New Orleans, Louisiana, USA.; ^2^ Ochsner BioDesign, Ochsner Academics, New Orleans, Louisiana, USA.; ^3^ Ochsner Health System, University of Queensland School of Medicine-Ochsner Clinical School, New Orleans, Louisiana, USA.; ^4^ Department of Pediatrics, Division of Critical Care, University of Alabama at Birmingham, Birmingham, Alabama, USA.

**Keywords:** medical simulation, medical education, augmented reality, mixed reality

## Abstract

**Background:**

The acquisition of procedural skills by medical trainees remains challenging, time-consuming, and risky. The use of augmented reality in the clinical setting is increasing because of its growing accessibility and potential to aid medical education and clinical care delivery.

**Objective:**

This study investigated the feasibility, adoption, and efficacy of augmented reality (AR)-supported lumbar puncture (LP) simulation for medical student training.

**Methods:**

A randomized and controlled approach was used to assess the effect of AR-supported simulation training versus standard simulation training on the number of sticks for successful LP, distance of first puncture from the target, and the time to successful LP. Pre- and post-surveys were used to assess medical student satisfaction with AR-supported LP simulation.

**Results:**

Thirty-four third-and fourth-year medical students were equally randomized into a AR-supported or control LP simulation groups. The median number of punctures required for a successful LP was 2 (interquartile range [IQR] 2) in the control group and 1 (IQR 2) in the AR group (*p* = 0.58). The median distance of the first puncture from the target was 5 mm (IQR 25) in the control group and 10 mm (IQR 18) in the AR group (*p* = 0.51). The median time to successful puncture was 137.9 s (IQR 283.1) in the control group and 93.8 s (IQR 117.7) in the AR group (*p* = 0.27). Confidence in performing LP on real patients on a 1–5 Likert scale improved from a median of 1 (IQR = 1) to 3 (IQR = 1, *p* < 0.01).

**Conclusions:**

There was no statistically significant difference in the number of sticks, distance from the target, and the time to successful puncture between the AR and control groups. Significant improvements in confidence levels were reported after simulation training via a survey. Although most of this cohort had no previous exposure to AR, the majority expressed willingness and preference to use AR in the procedural simulation. Future studies would benefit from a multisite design for a larger sample, as well as the use of newer AR technologies.

## Background

Augmented reality (AR) was first introduced in the early 1990s and is now an important tool in industries such as automotive manufacturing, aerospace, and video gaming. Unlike virtual reality (VR), which completely immerses users in a digital environment, AR juxtaposes a virtual image, i.e., a hologram, in a real-life setting. The recent release of consumer-grade AR head-mounted displays (HMD), i.e., headsets, has expedited adoption in a diverse array of fields including health care.^1,2^ AR is increasingly being used in patient education, therapy, preoperative planning, and procedural execution in clinical fields ranging from spine disease, orthopedics, and critical care medicine.^3–8^ AR has also been used as a treatment modality in disease states like Parkinson’s disease, vestibulopathies, and post-traumatic stress syndrome.^9–11^ Numerous investigations into the benefits of AR in clinical education, demonstrating the technology’s ability to supplement current learning methods have been undertaken.^12–17^ Medical trainees using three-dimensional AR models develop a better understanding of spatial complexity of different anatomical structures and relationships, with reduced cognitive load and increased interest/engagement in learning.^12–14,18,19^

Traditional approaches to procedural training in medical school and residency entail a significant amount of pre-patient training, such as didactics, videos, and skills practice with feedback to diminish the risk to patients and optimize *in vivo* learning opportunities. Obtaining the requisite psychomotor skills for medical procedures should leverage a diverse array of modalities ranging from cadavers, task trainer simulators, and mixed reality.^20^ AR holds promise as another platform for medical simulation and procedural skills acquisition in the pre-patient training phase. Emerging uses of AR for medical procedural simulation include emergency triage by paramedics, training for invasive bedside procedures, and teaching of surgical skills.^21–27^ By pairing AR with current simulation trainers, academic and nonacademic medical institutions can improve procedural preparedness by supporting readily available, relatively low-cost, and risk-free simulation.^28^

Lumbar punctures (LPs) are invasive procedures that allows for cerebrospinal fluid analysis. They are relatively safe but are often associated post-procedural headaches and less often with more serious complications like infection, highlighting the importance of precision and skill in their execution.^29^ In practice, their complexity can vary owing to significant anatomical variation and difficulty visualizing anatomical landmarks.^30^ AR has the potential to address both educational and clinical training needs given the procedure’s frequent inclusion in simulation training and the broad range of clinicians who perform LPs. A recent nonrandomized uncontrolled trial demonstrated the feasibility of using extended reality in LP simulation.^31^

Our study’s research questions focused on the utility and adoption of AR in LP simulations when compared with standard simulations. The population of interest was third- and fourth-year medical students who had never performed LPs. The intervention was an AR HMD-based spine visualization application, which was compared with a standard task trainer-based LP simulator. Our primary outcome was the number of sticks required to successfully elicit fluid from the LP simulator. Secondary outcomes included the distance of the first puncture from the target and time to successful puncture. Furthermore, surveys were used to assess the willingness of the study cohort to utilize AR in future simulation training and to elicit general feedback about the use of AR in procedural training. We hypothesized that the use of an AR-enhanced LP simulation would reduce the number of sticks required for a successful LP in a simulation model when compared with standard simulation alone.

## Materials and Methods

This study was approved by the institutional review board of Ochsner Health with a consent waiver. An information sheet outlining the study details was provided to all subjects. Disclosures were obtained for any recognizable person in the photographs. Data not included in this article were stored and are available upon request.

This study was performed at the Ochsner Clinical Simulation and Patient Safety Center in New Orleans, LA. The materials used included a Microsoft^Ⓡ^ Hololens (Redmond, WA) HMD, Simulab^Ⓡ^ Lumbar Puncture Trainer (Seattle, WA), and 20-gauge spinal needles. A Microsoft Hololens AR spine hologram registration application was developed by the Ochsner BioDesign Lab utilizing a human-centered design approach focused on the experience of clinicians and trainees, as described in the first phase of the VR Clinical Outcomes Research Experts model.^32^ The application utilizes physical fiducial markers to allow fine control of the spine model placement, allowing separate input for positioning and per-axis rotation of the holographic spine. The initial position was achieved by placing a primary fiducial marker on top of the LP task trainer. Additional adjustment of a second fiducial marker allowed for rotational adjustment. To reduce positioning errors, a dynamic confidence algorithm was used to track the markers and reduce jumps from false detections. This was achieved by aggregating confidence while stationary and ignoring implausible movement speeds typical of false detections from distant surfaces. Once properly positioned, the spine was locked into place, whereupon the fiducials were removed.

**FIG. 1.**
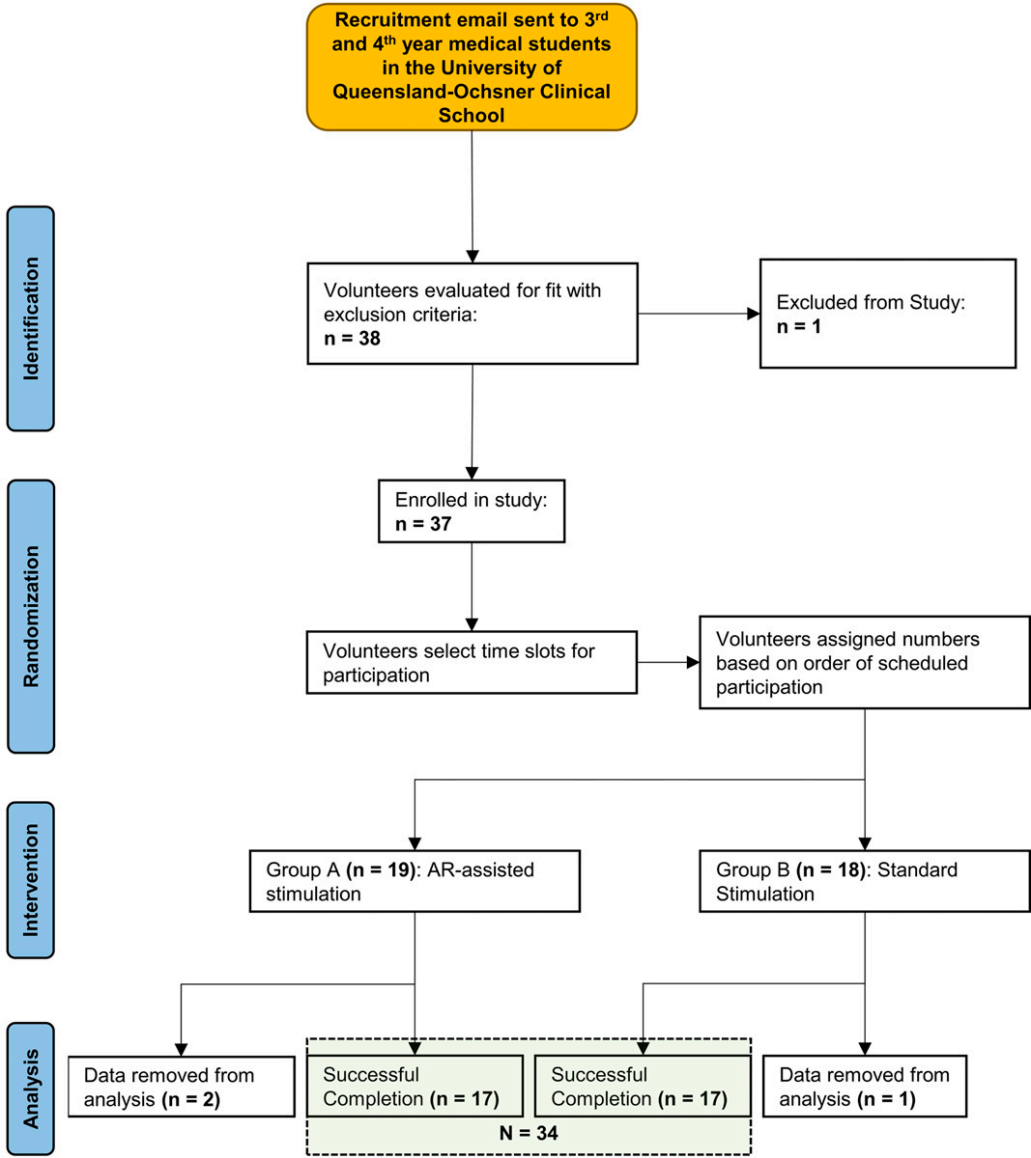
Participant flow.

**FIG. 2.**
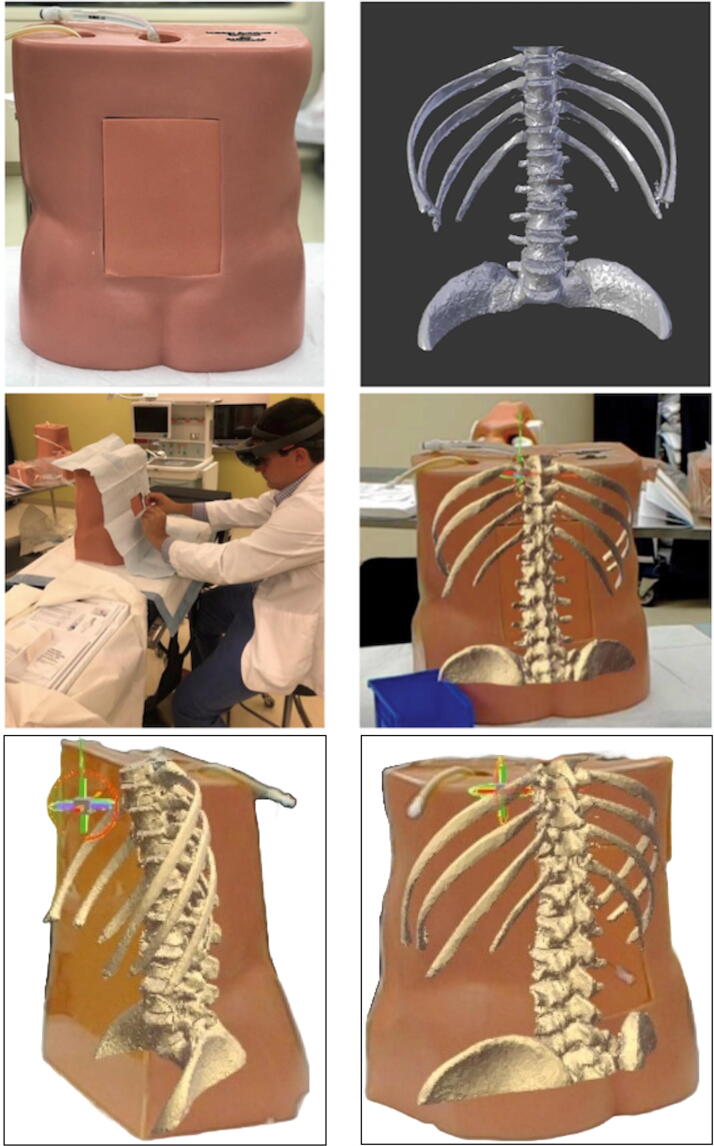
Experimental setup with augmented reality head mounted display Upper left: Simulab^Ⓡ^ Lumbar Puncture Simulator. Upper right: Spine hologram rendered from computed tomography (CT) imaging. Middle left: Student attempting procedure with AR-assisted co-registered hologram. Middle right and Lower right: Spine hologram (coronal view) projected on to simulator as seen via AR HMD. Lower left: Spine hologram in sagittal view. AR, augmented reality; AR HMD, augmented reality head-mounted displays.

Third- and fourth-year medical students from the University of Queensland-Ochsner Clinical School were recruited via e-mail at the beginning of the academic year over a 3-month period via email. Students who had previously performed LPs, either on live patients or as part of simulation training, were excluded from the study. This study utilized a randomized approach in which subjects performed LPs with or without AR assistance (Fig. 1). Subjects in the control group performed LP without AR assistance, whereas those in the intervention group performed LP using AR assistance (Fig. 2). Randomization was performed using GraphPad Software, an online randomization tool (https://www.graphpad.com/quickcalcs/randomize1.cfm). All subjects completed a demographic survey as well as pre- and post-study Likert-scale questionnaires to explore attitudes about the procedure and the use of AR in medical simulation.

After completing the questionnaire, subjects watched an instructional video on proper technique for performance of an LP done by the traditional method via palpation of anatomical landmarks.^33^ Subjects then completed a brief tutorial on the HoloLens AR HMD and the Ochsner Augmented Reality Lumbar Puncture application. The research fellow (J.C.) co-registered and confirmed placement of the spine hologram on the simulator using a predetermined, internally developed method. This method included the use of fiducials to ensure consistent placement of the hologram and reduce variability in the experimental design. While wearing the HMD, subjects were provided a 5-min tutorial on how to adjust the hologram’s transparency and how to visualize their hands and the needle while performing the LP with the HMD. At this point, subjects could change the transparency of the hologram but could not make other changes to the spine hologram’s size, location, and orientation.

For each trial, a new piece of fabric was placed over the center of the task trainer to determine the exact location of the first puncture relative to the ideal target. For the AR group, subjects were instructed to place the HMD on their heads and confirm their ability to visualize the spine hologram on the task trainer. Individuals who were randomized to AR also completed a second trial without AR; conversely, those initially randomized to the control group completed a second trial with AR. Post-survey data were collected via e-mail within 1 week after the completion of these trials.

### Statistical plan

The participant characteristics examined in the analysis are listed in Table 1 and included subject demographics (age, sex, year of medical school, race) as well as previous exposure to AR. The statistical plan for this study was designed to analyze the effectiveness of AR in teaching LP procedures to medical students. The method of LP, with or without AR, was the independent variable. The outcome variables collected included the number of punctures required to elicit fluid successfully, distance from the ideal target in the L4/L5 interspace of the first stick, and time to successfully elicit fluid from the simulator representing a successful LP. The Shapiro–Wilk test was used to assess the normality of the collected data to determine the appropriate parametric or nonparametric statistical tests. The median number of sticks, distance, and time with the corresponding interquartile range (IQR) was calculated. The Mann–Whitney U test and the Wilcoxon Signed Rank test were used to compare the number of sticks to successful tap, distance of first puncture from the L4/L5 target space, time to successful tap, and Likert Scale-based survey data.

**Table 1.** Demographics and Characteristics of Medical Student Cohort

Variable	Control (n = 17)	AR (n = 17)	Total (N = 34)	Statistical test (control vs. AR)
Ethnicity	Asian: 18.75%	Asian: 11.76%	Asian: *n* = 5 (14.7%)	N/A
	Caucasian: 50%	Caucasian: 88.24%	Caucasian: *n* = 23 (67.7%)	
	Hispanic: 6.25%		Hispanic: *n* = 1 (2.9%)	
	Indian: 12.50%		Indian: *n* = 2 (5.9%)	
	Other: 6.25%		Other (N/A, Missing, Other): *n* = 3 (8.8%)	
Gender	Female (F): 41.18%	Female (F): 35.29%	Female: *n* = 13 (38.2%)	Chi-squared
	Male (M): 58.82%	Male (M): 64.71%	Male: *n* = 21 (61.8%)	(*p* = 1)
Year	Year 3: 17.65%	Year 3: 35.29%	Year 3: *n* = 9 (26.5%)	Chi-squared
	Year 4: 82.35%	Year 4: 64.71%	Year 4: *n* = 25 (73.5%)	(*p* = 0.437)
Age	Mean: 28.47 years	Mean: 28.18 years	Mean: 28.32 years	Mann–Whitney U
	Median: 27.00 years	Median: 28.00 years	Median: 28.00 years	(*p* = 0.793)
Used AR before	Yes: 11.76%	Yes: 35.29%	Yes: *n* = 8 (23.53%)	Fisher’s exact
	No: 88.24%	No: 64.71%	No: *n* = 26 (76.47%)	(*p* = 0.229)
LPs Seen	Mean: 3.82	Mean: 5.06	Mean: 4.44	Mann–Whitney U
	Median: 3.0	Median: 2.0	Median: 2.5	(*p* = 0.766)

Comparison of demographic and behavioral variables between the control group (n=17) and the augmented reality (AR) group (n=17), including ethnicity distributions, gender ratios, academic year distribution, age statistics, previous experience with augmented reality, and the number of LPs seen. No significant differences were found between the two groups for any of the variables listed.

AR, augmented reality; LP, lumbar puncture.

## Results

A convenience sample of 37 medical students was recruited over a 3-month period at the beginning of the academic year. Data from three of these subjects were discarded because of technical difficulties with the simulator and/or the HMD. Specifically, the spine hologram disappeared from the HMD’s field of view during the procedure attempt. The data from the remaining 34 subjects were analyzed for the number of punctures required to elicit fluid successfully and the average distance of the first puncture from what the ideal needle puncture location in the L4/L5 interspace. This target was the center of an area, 10 mm long × 5 mm wide, spanning the top of the L5 spinous process to the middle of the L4/L5 interspace on the LP task trainer.

The average age of the subjects was 28.3 years (range 24–41). Fourth-year medical students accounted for 73.5% of the subjects, and the remainder were third-year medical students; 61.8% were male, and 76.5% had not been exposed to AR before this study (Table 1). An analysis of the association between previous use of AR and group assignment (AB or BA) was conducted with a Fisher’s exact test due to the presence of expected frequencies <5 in some cells of the contingency table, which made it a more appropriate choice over the chi-square test of independence. The dataset comprised observations from 32 individuals, categorized based on their previous AR usage (Yes or No) and their AB or BA. The null hypothesis posited that there was no significant association between having used AR before and the group assignment for which the Fisher’s exact test yielded a nonsignificant *p* value of 0.229.

**FIG. 3.**
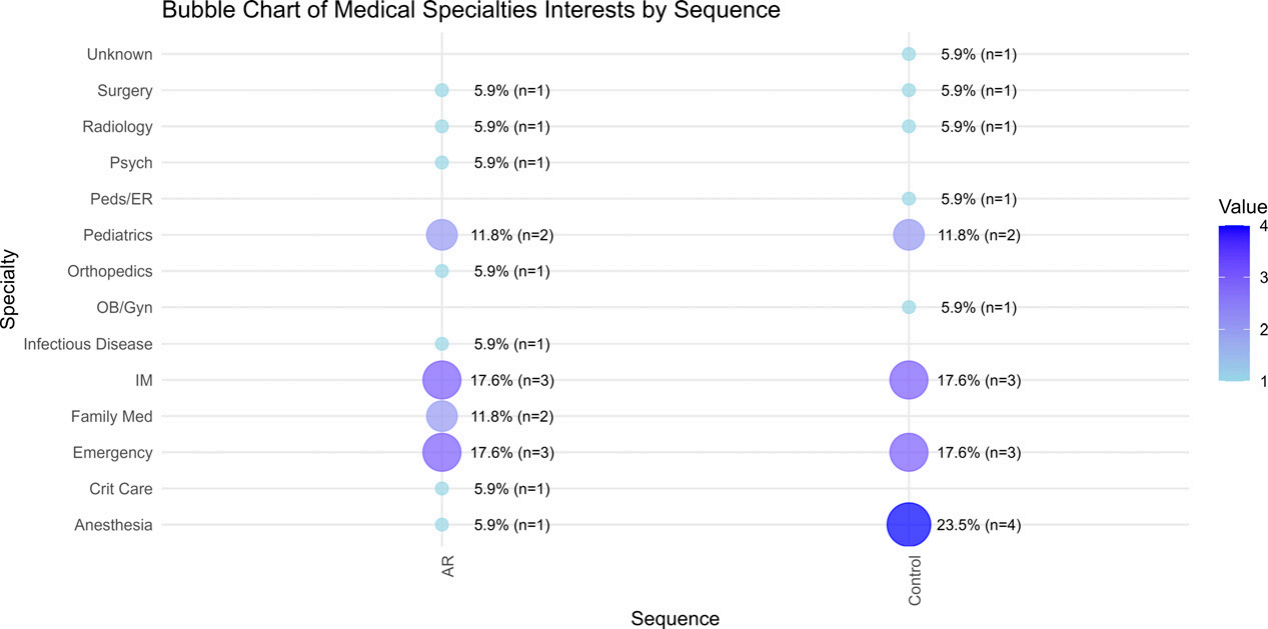
Visualization of the specialty interests of the participating medical students.

The medical student participants reported interest in a heterogenous set of specialties (Fig. 3). The comparison of demographic and behavioral characteristics, including ethnicity, gender, academic year, age, previous experience with AR, and the number of LPs observed, revealed no significant differences between the groups. Statistical testing of these attributes between groups yielded nonsignificant *p* values indicating successful randomization and stratification in participant allocation (Table 1).

The Shapiro–Wilk test for normality was used to assess the distribution of the outcome data and inform the selection of the appropriate statistical test. For the number of punctures required for successful LP, the Shapiro–Wilk test for normality was significant for both the AR group (W = 0.6188, *p* < 0.01) and the control group (W = 0.7212, *p* < 0.01). Similarly, for the distance of the first puncture from the target, the Shapiro–Wilk test statistic was significant for the AR group (W = 0.8562, *p* < 0.01) and the control group (W = 0.7916, *p* < 0.01). Finally, the time to successful puncture showed a statistically significant Shapiro–Wilk test statistic for the AR group (W = 0. 8639, *p* < 0.01), and control groups (W = 0.8541, *p* < 0.01). These results indicate that the number of punctures, distance of first puncture from the target, and time to successful puncture did not follow a normal distribution in either the control or AR group. Thus, nonparametric statistical tests, such as the Mann–Whitney U test for between-group and Wilcoxon-signed rank test for within-group analyses, were utilized in the analysis.

**FIG. 4.**
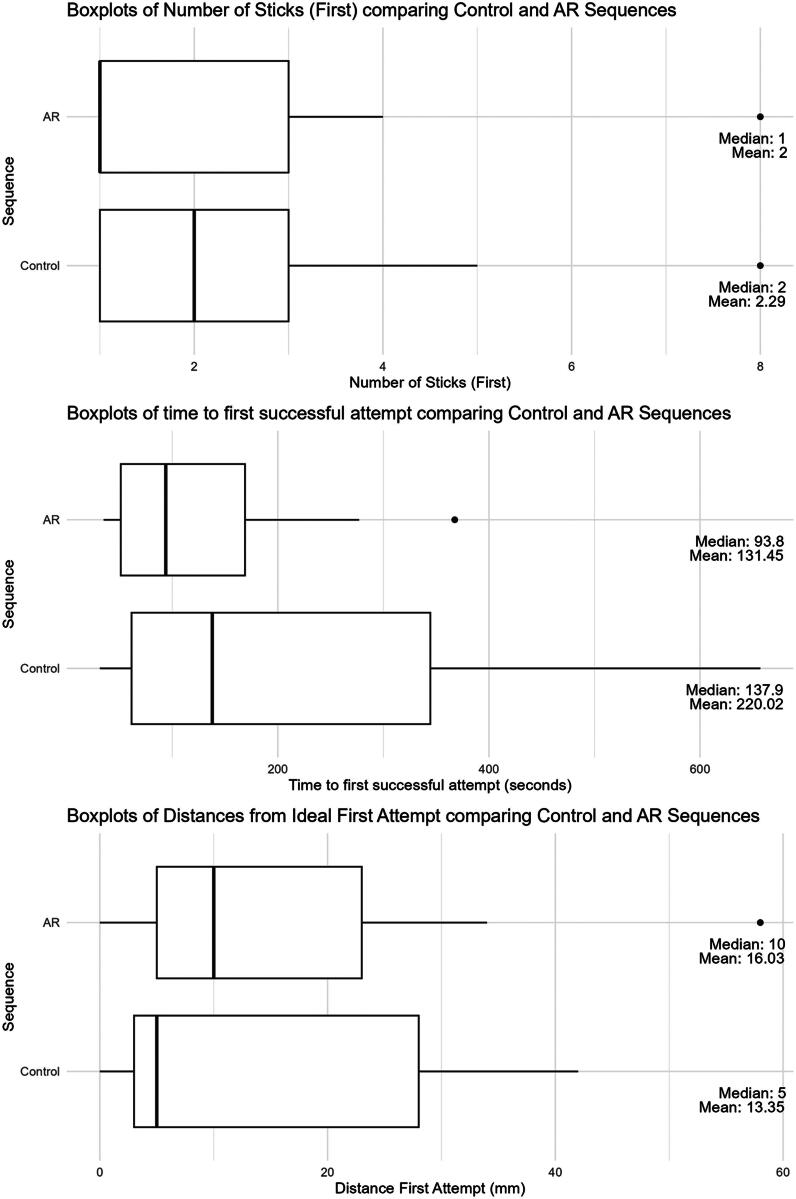
Boxplots comparing sequences control (AB) and AR-assisted (BA) for number of sticks, time to first successful attempt and distances from ideal first attempt.

The number of punctures required to elicit fluid was the primary outcome measure assessed (Fig. 4). The median number of punctures required for successful LP was 2 (IQR 2) in the control group and 1 (IQR 2) in the AR group, with no statistically significant difference between the groups (*n* = 34, W = 165, *p* = 0.58), The median distance of the first puncture from the target between the L4 and L5 vertebral spaces, a secondary outcome, was 5 mm (IQR 25) in the control group and 10 mm (IQR 18) in the AR group (*n* = 34, W = 125, *p* = 0.51), which also was not significantly different between groups. The median time to successful puncture was 137.9 s (IQR 283.1) in the control group and 93.8 s (IQR 117.7) in the AR group and was not significantly different between groups (*n* = 34, W = 177, *p* = −0.27).

Within-group analysis was also performed to determine if the order affected the number of punctures required, time to successful fluid elicitation, and distance from ideal target. The findings demonstrated a significant difference in the time to successful fluid elicitation in the control then AR AB sequence (*p* < 0.01) with a median time decrease from 137.9 s in the first trial to 45.8 s in the second. Conversely, in the AR first and the control BA sequence, no significant difference in time was noted (*p* > 0.05) with median times of 93.8 s initially and 68.8 s. Additional with-in group analysis did not reveal significant differences for the number of punctures required and distance from ideal target.

**FIG. 5.**
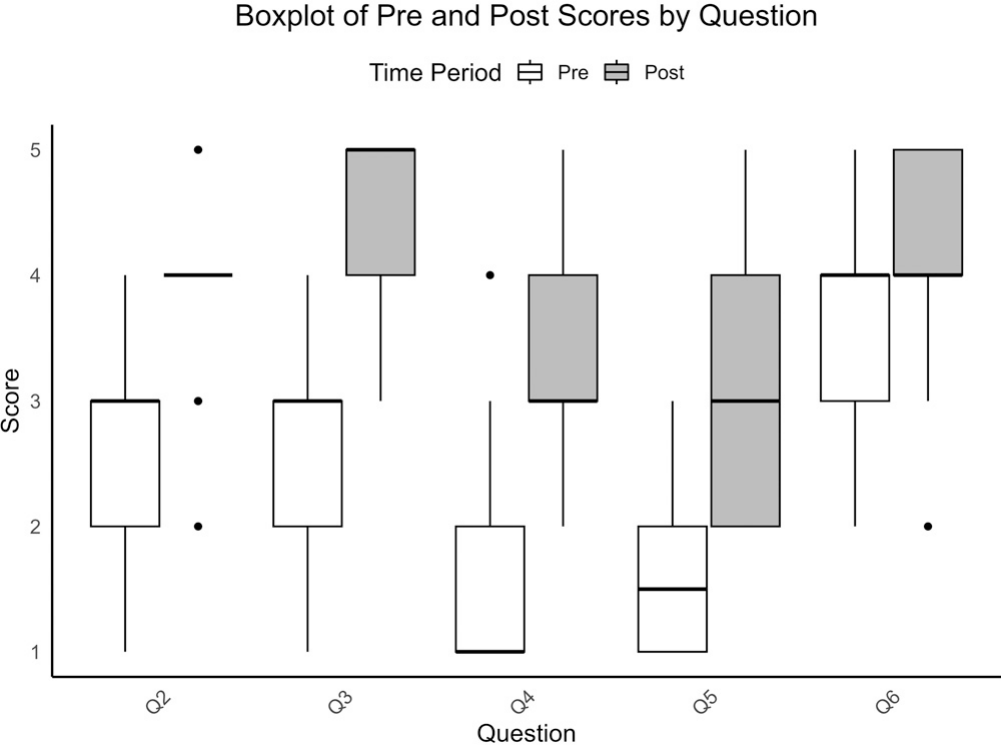
Distribution of pre- and post-intervention responses for survey questions Q2 to Q6 on a 1–5 Likert-based scale, where 1 represents “strongly disagree” and 5 represents “strongly agree.” The questions evaluate participants’ confidence in various aspects of performing a lumbar puncture, including locating the needle insertion site (Q2), on a practice dummy (Q3), on a real patient (Q4), without instructor guidance (Q5), and the perceived benefit of augmented reality guidance (Q6). Boxplots show the response range, with Wilcoxon signed-rank test results provided for each question.

The subjects were asked to complete pre- and post-study questionnaires investigating attitudes towards their ability to perform LPs. A 1–5 Likert-based scale was used, with responses ranging from strongly disagree (1) to strongly agree (5). To accurately conduct the Wilcoxon signed-rank test, it was essential to verify that every pre- and post-pair for each question comprised an equal number of complete data pairs, with no missing values. Of the 34 post-simulation surveys sent electronically, 32 were completed; thus, two pairs were not included in the analysis. Of note, surveys were completed after all subjects, including controls, were exposed to the LP simulation with AR (Fig. 5). For Q2 regarding confidence in the needle insertion site to perform an LP, the median score and IQR were 3 and 1 before simulation and 4 and 1 after simulation, respectively (*n* = 32, V = 0, *p* < 0.01). For Q3 regarding confidence in successfully performing an LP with a task trainer, the median score and IQR were 3 and 1 before simulation and 5 and 1 after simulation, respectively (*n* = 32, V = 0, *p* < 0.01). For Q4 regarding confidence in successfully performing an LP in a real patient, the median score and IQR were 1 and 1 before simulation and 3 and 1 after simulation, respectively (*n* = 32, V = 0, *p* < 0.01). For Q5 regarding confidence in successfully performing an LP without instructor guidance, the median score and IQR were 1.5 and 1 before simulation and 3 and 2 after simulation, respectively (*n* = 32, V = 0, *p* < 0.01). For Q6, where subjects were asked if they would feel more comfortable in their ability to perform an LP if given AR guidance, the median score and IQR were 4 and 1 before simulation and 4 and 1 after simulation, respectively (*n* = 32, V = 21, *p* = 0.02). In the analysis of survey responses pre- and post-intervention, Wilcoxon signed-rank tests revealed statistically significant differences for all questions (Q2: V = 0, *p* < 0.01; Q3: V = 0, *p* < 0.01; Q4: V = 0, *p* < 0.01; Q5: V = 0, *p* < 0.01; Q6: V = 21, *p* = 0.02).

## Discussion

This pilot study demonstrated the feasibility of using AR HMDs to assist in LP simulation training. There were no significant differences in the metrics of efficacy between the AR and control groups, specifically the number of punctures, mean distance of the first puncture, and time to successful simulated puncture. Finally, the survey data showed *a priori* willingness by medical students to adopt AR in procedural training, which increased after using of the technology.

This study had several limitations. Although randomized and controlled, the sample size was small, and neither the subjects nor the research fellow was blinded to the intervention. The control group did not include a sham, but rather the absence of the intervention. Surveys were completed after all the subjects had the opportunity to use AR in the LP simulation. Thus, the impact of AR on increased confidence reflected in the survey responses could not be disambiguated from the impact of exposure to standard simulation. The results of the Fisher’s exact test, however, suggests that within the limits of this study, there is no significant evidence to indicate an association between prior AR use and group assignment. We thus conclude that our primary and secondary outcomes were not confounded by differences in previous AR experience and cohort assignment.

Of note, a significant reduction in time to successful LP was found in the cohort who first did the control and then AR-enhanced simulation. The AR to traditional simulation group also showed a decrease in median time to successful puncture but this change did not achieve statistical significance. These findings suggest that AR may enhance procedural efficiency when integrated with traditional simulation.

While the AR HMD technology worked relatively dependably, more development is needed to further improve their user interface and reliability. Additionally, 14 subjects commented on the heaviness and bulkiness of the headset and its limited field of view. These issues are well documented with current AR HMD technologies.^34^ Newer headsets with more advanced and robust functionalities and improved user interfaces will reduce these barriers.

There were no statistically significant differences in the primary and secondary outcomes between the AR and control groups. Although the AR group did not demonstrate statistically significantly improved proficiency in LP simulation, neither did this novel technology result in worse performance. Finally, the results of the statistical testing may be due to the small sample size rather than the absence of AR utility in the procedural simulation.

The survey data results strongly suggest significant shifts in respondent attitudes and perceptions associated with the use of AR in the LP simulation. Survey responses demonstrated an increase in confidence in the ability to locate the LP insertion site, perform an LP on a task trainer successfully, and perform an LP on a patient successfully, and without an instructor, after the completion of the study (Fig. 4). Analysis of participants’ attitudes concerning the benefits of AR assistance in LP training demonstrated an *a priori* desire to use AR in procedural training that persisted after exposure. This finding warrants further exploration into the specific nature and character of these changes. The usability and perceived utility of novel technologies can be a practical barrier to the adoption of new approaches in medical training and care delivery.^35–37^ As noted by multiple subjects; however, there was significant enthusiasm for the use of AR in procedural simulation in this cohort as well as confidence in its utility despite the suboptimal usability of a first-generation AR HMD.

### Interpretation and future directions

Technologies such as AR, which minimize patient risk and optimize simulation realism, are expected to play a growing role in medical education, simulation, and training. Tools to facilitate pre-patient training are particularly important for the next generation of clinicians training in the midst and aftermath of the COVID Pandemic. Even though most subjects had never been exposed to AR, the high average pre-study score confirmed the willingness of medical students to adopt this technology as a tool for procedural training. Moreover, this study suggests that students who used AR for this training believed it would improve their confidence when performing an LP. The need to learn and use a novel technology, such as AR HMD’s, did not diminish their enthusiasm for the technology. To the contrary, students showed great enthusiasm for adopting and incorporating this technology into their training. Post-study feedback confirmed pre-study engagement (32 of 34 eligible participants responded to the post-study questionnaire), with most students telling the researchers that they found the AR hologram highly useful when they attempted to perform an LP. The medical student subjects were willing and eager to participate in a study using a novel mixed reality tool. This enthusiasm will facilitate further study and generalizability of AR in medical simulation in other medical procedures.

There are several ways future studies could leverage this enthusiasm and address limitations of the current study. A recent study found that spaced VR simulator training enhances surgical skill acquisition compared with massed (single day) training.^38^ Future study designs would benefit from repeated or serial sessions to characterize improvements in accuracy, efficiency, and confidence among medical students over time. Future studies would ideally not only assess AR’s utility in LP simulation but also *in vivo.*

Traumatic and failed LPs can be associated with adverse effects resulting in suboptimal patient outcomes and necessitate repeat and more resource-intensive procedures, e.g., fluoroscopy-guided LPs.^29^ Improved performance of LPs has been associated with reduced risk of both traumatic and failed LP’s.^39^ Improved LP performance by both experts and novices can lead to improved patient experience and outcomes.

There may also be a relationship between AR’s utility in simulation and the complexity, anatomical variability, and associated frequency of the procedure. Thus, the impact of AR on a relatively standard and frequent procedure like LP simulation may be difficult to detect when compared with traditional and accessible simulation. Future studies assessing AR’s value in simulation may consider focusing on more complex, infrequent, and/or highly variable procedures without standard and accessible simulation paradigms where the impact of AR compared may be more profound. Specifically, AR may enhance training and outcomes in complex medical procedures, such as those performed in neurosurgery, congenital cardiac interventions, and orthopedic surgery.

More sophisticated and enterprise AR HMDs are being released with improved capabilities that will minimize the risk of technical failures, decrease the barriers to adoption by clinical trainees, as well as expand the potential applications of AR in care delivery. The effect of AR on speed and quality of skill development requires further evaluation. Future studies should incorporate improved HMDs to rigorously assess AR’s utility in improving procedural proficiency and competency.

This study demonstrated the eagerness of medical trainees to use novel technologies such as AR in their education and provided preliminary framework to assess its utility. We anticipate that AR will have a growing role and impact on medical education and health care in general.

## Abbreviations Used

AR: Augmented Reality
HMD: Head-Mounted Display
LP: Lumbar Puncture
VR: Virtual Reality
